# Serum *Wisteria floribunda* agglutinin-positive Mac-2-binding protein levels predict the presence of fibrotic nonalcoholic steatohepatitis (NASH) and NASH cirrhosis

**DOI:** 10.1371/journal.pone.0202226

**Published:** 2018-08-30

**Authors:** Naim Alkhouri, Casey Johnson, Leon Adams, Sachiko Kitajima, Chikayuki Tsuruno, Tracey L. Colpitts, Kazuki Hatcho, Eric Lawitz, Rocio Lopez, Ariel Feldstein

**Affiliations:** 1 The Texas Liver Institute, University of Texas (UT) Health Science Center, San Antonio, Texas, United States of America; 2 Department of Pediatrics, University of California – San Diego, La Jolla, California, United States of America; 3 The University of Western Australia, Crawley, Australia; 4 Sysmex Corporation, Kobe, Japan; 5 Sysmex Corporation R&D Center of Americas, Lincolnshire, Illinois, United States of America; 6 Digestive Disease and Surgery Institute, Cleveland Clinic, Cleveland, Ohio, United States of America; Medizinische Fakultat der RWTH Aachen, GERMANY

## Abstract

**Objective:**

The race for finding effective treatments for nonalcoholic fatty liver disease (NAFLD) has been slowed down by the high screen-failure rate for including patients in trials due to the lack of a noninvasive biomarker that can identify patients with significant disease. Recently, *Wisteria floribunda* agglutinin-positive Mac-2-binding protein (WFA^+^ -M2BP) has shown promise in predicting liver fibrosis. The aims of this study were to evaluate the utility of WFA^+^ -M2BP as a biomarker to sub-classify patients with NAFLD according to their disease severity and to assess its correlation with histologic features of NAFLD.

**Methods:**

Patients undergoing biopsy for clinical suspicion of NAFLD and healthy controls were included. Patients with NAFLD were classified into: NAFL, early NASH (F0-F1), fibrotic NASH (F2-F3), and NASH cirrhosis (F4). Levels of WFA^+^ -M2BP in sera was measured by a HISCL^™^ M2BPGi^™^ assay kit using an automated immunoanalyzer (HISCL^™^-800; Sysmex, Kobe, Japan). Analysis of covariance was used to assess difference in WFA^+^ -M2BP between the groups and Spearman’s correlation coefficients were used to assess correlation with histological features.

**Results:**

Our cohort consisted of 20 healthy controls and 198 patients with biopsy-proven NAFLD divided as follows: 52 with NAFL, 62 with early NASH, 52 with fibrotic NASH, and 32 with NASH cirrhosis. WFA^+^ -M2BP level was found to be significantly increased in the fibrotic NASH and NASH cirrhosis groups compared to healthy controls and those with early NAFLD after adjusting for age, gender and BMI. Furthermore, patients with NASH cirrhosis had significantly higher WFA^+^ -M2BP levels (2.4[1.5, 4.2] C.O.I (Cut-off Index)) than those with fibrotic NASH (1.2[0.79, 1.9]), p < 0.001. WFA^+^ -M2BP level had moderate correlation with inflammation, ballooning and NAFLD activity score and strong correlation with fibrosis stage. Additionally, ROC curve analysis demonstrated that WFA^+^ -M2BP accurately differentiated F2-4 from F0-F1.

**Conclusion:**

In a large cohort of patients with the full spectrum of NAFLD, WFA^+^ -M2BP levels predicted the presence of advanced disease and correlated strongly with fibrosis stage.

## Introduction

Although the initial description of nonalcoholic fatty liver disease (NAFLD) dates back to 1980 [1], its full spectrum and impact as a major chronic liver disease has only been recognized for the past 2 decades [2]. Today, NAFLD is a leading indication for liver transplantation [3, 4] and contributes as a major underlying etiology for hepatocellular carcinoma (HCC) in the United States [5].

NAFLD includes a histologic spectrum of diseases that starts with bland accumulation of fat within the hepatocytes without evidence of significant necro-inflammation or fibrosis, a condition called nonalcoholic fatty liver (NAFL) [6]. Nonalcoholic steatohepatitis (NASH) is considered the aggressive form with evidence of liver inflammation and hepatocyte injury in the form of ballooning. NASH is the driving force behind the development of significant liver fibrosis, the most important prognostic factor in predicting liver-related outcomes [7, 8]. Finally, the development of NASH cirrhosis, which occurs in 10–25% of patients with fibrotic NASH, has significant clinical implications in terms of the development of end-stage liver disease requiring transplantation and HCC [9]. Therefore, fibrotic NASH is a serious illness that warrants aggressive medical management with different pharmacologic agents given the serious limitations of lifestyle modifications in achieving and sustaining meaningful weight loss [10]. Drugs that target specific pathways that play a role in NAFLD development and progression are being developed at a rapid pace. Unfortunately, liver biopsy remains the gold standard for differentiating between NAFL/ early NASH and fibrotic NASH in addition to providing information regarding the degree of steatosis, severity of inflammatory activity and stage of fibrosis. This need for biopsy/ histology to determine which patients require treatment and assess their response to therapy has slowed down the race for finding effective treatments for NAFLD due to patient hesitation to undergo several biopsies and the high screen-failure rate for including patients in clinical trial. Therefore, there is an urgent need to develop a simple noninvasive biomarker that can classify patients into those with early disease and those with advanced disease that will benefit the most from pharmacologic treatment.

Several recent studies have shown that *Wisteria floribunda* agglutinin-positive Mac-2 binding protein (WFA^+^ -M2BP) is a promising biomarker in predicting the severity of liver fibrosis in different chronic liver diseases [11]. Mac-2 (galectin-3) binding protein (M2BP) is a glycoprotein that is almost undetectable in normal liver but becomes easily detected in patients with hepatocyte injury as liver fibrosis progresses. WFA^+^ -M2BP can distinguish the glycan structure of WFA-detectable M2BP and therefore is considered a biomarker for liver injury and fibrosis. Although a few studies have evaluated the use of WFA^+^ -M2BP to assess the severity of NAFLD, they were exclusively done in Asian populations with no available data to our knowledge in other ethnic/ racial groups. Furthermore, the main focus has been on utilizing WFA^+^ -M2BP to predict fibrosis stage and not the other histologic feature or the presence of fibrotic NASH.

The aims of this study were to evaluate the utility of WFA^+^ -M2BP as a biomarker to sub-classify a diverse group of Western patients with NAFLD according to their disease severity and to assess correlation between WFA^+^ -M2BP and individual histologic features of NAFLD (steatosis, ballooning, and inflammation, and fibrosis).

## Methods

### Subjects and histological diagnosis

Patients undergoing liver biopsy for clinical suspicion of NAFLD and healthy controls (age- and gender-matched lean individuals with no components of the metabolic syndrome) were included. Informed consent was obtained from all subjects and the study was approved by the institutional review board at the University of Western Australia, Crawley, Australia. The histological diagnosis was established using hematoxylin-eosin and Masson trichrome stains of formalin-fixed paraffin-embedded liver and graded by an experienced hepatopathologist who was blinded to the clinical characteristics of the patients. The hepatopathologist provided an overall diagnostic interpretation and also reported a NAFLD Activity Score (NAS) for each patient based on the NAFLD scoring system proposed by the National Institute of Diabetes and Digestive and Kidney Diseases NASH Clinical Research Network [12]. According to this scoring system, the degree of steatosis and inflammatory activity is measured using a 9-point scale (steatosis = 0–3; lobular inflammation = 0–3; ballooning = 0–2). The NAS is the unweighted sum of steatosis, lobular inflammation, and hepatocellular ballooning scores. The stage of fibrosis was measured using a 5-point scale (F0 = no fibrosis, F1 = perisinusoidal or portal/periportal fibrosis; F2 = perisinusoidal and portal/periportal fibrosis; F3 = bridging fibrosis; F4 = cirrhosis).

Patients with NAFLD were classified according to EASL guidelines [13] into the following: NAFL, early NASH (F0-F1), fibrotic NASH (F2-F3), and NASH cirrhosis.

### Clinical and biochemical data

Clinical data including age, sex, and body mass index (BMI) were documented for all patients at the time of liver biopsy. Blood samples obtained from patients at the time of their liver biopsies were initially processed to serum then stored frozen at– 80 °C in our bio-bank. Complete blood count and liver biochemistry parameters were measured using a conventional automated analyzer including serums aspartate aminotransferase (AST), alanine aminotransferase (ALT), alkaline phosphatase, total bilirubin, and platelet count. The AST-to-platelet ratio index (APRI) was calculated as AST/ULN (upper limit of normal)/Platelets x 100. The FIB-4 index was calculated using the following formula: FIB-4 = (Age x AST)/ [Platelet count (10.9/L) x √ALT] [14]. Level of WFA^+^ -M2BP in sera was measured by a HISCL^™^ M2BPGi^™^ assay kit using an automated immunoanalyzer (HISCL^™^-800; Sysmex, Kobe, Japan). The measured values of WFA^+^ -M2BP conjugated to WFA were indexed with obtained values using the following equation: Cut-off index (C.O.I) = ([WFA^+^ -M2BP]sample–[WFA^+^ -M2BP]_NC_) / ([WFA^+^ -M2BP]_PC_−[WFA^+^ -M2BP]_NC_), where [WFA^+^ -M2BP]sample was the WFA^+^ -M2BP count of the serum samples, PC was positive control and NC was negative control. The positive control was supplied as a calibration solution preliminarily standardized to yield a cut-off value of 1.0 (13).

### Statistical analysis

Data are presented as mean ± standard deviation, median [25th, 75th percentiles] or frequency (percent). A univariable analysis was performed to assess differences between the 5 diagnosis groups (Healthy Controls, NAFL, early NASH, fibrotic NASH and NASH cirrhosis); analysis of variance (ANOVA) or the non-parametric Kruskal-Wallis tests were used to assess differences in continuous or ordinal variables and Pearson’s chi-square tests were used for categorical factors. In addition, analysis of covariance (ANCOVA) was used to assess differences in WFA^+^ -M2BP between the groups while adjusting for age, gender and BMI. The natural logarithm of WFA^+^ -M2BP was modeled as the outcome variables with diagnosis, age, gender and BMI as the independent predictors. All post-hoc comparisons were done using Bonferroni correction. Spearman’s correlation coefficients were used to assess correlation between WFA^+^ -M2BP and ALT as well as histologic characteristics of NAFLD. Lastly, Receiver Operating Characteristics (ROC) analysis was performed to assess the accuracy of WFA^+^ -M2BP in the diagnosis of fibrotic NASH in comparison to previously validated noninvasive fibrosis scores (APRI and FIB-4). SAS (version 9.4, The SAS Institute, Cary, NC) was used for all analyses and a p < 0.05 was considered statistically significant.

## Results

### Demographic and clinical characteristics

Our cohort consisted of 20 healthy controls and 198 patients with NAFLD: 52 with NAFL, 62 with early NASH, 52 with fibrotic NASH, and 32 with NASH cirrhosis. Table 1 presents a summary of subject characteristics including liver histology in those with NAFLD. Patients in the NAFL group (45.8 ± 11 years) were significantly younger than those in the fibrotic NASH (54.8±12.3 years) and NASH cirrhosis (56.1±6.7 years) groups; p value <0.001. There was no difference in gender distribution among the 5 groups. As expected, the control group had significantly lower mean BMI compared to the NAFLD groups, p value <0.001.

**Table 1:** Demographic and clinical characteristics.

Factor	Healthy Control (N = 20)	NAFL (N = 52)	Early NASH (N = 62)	Fibrotic NASH (N = 52)	NASH Cirrhosis (N = 32)	p-value
Age (years)	52.2±12.3	45.8±11.0 ^4^^,^^5^	50.2±11.9	54.8±12.3 ^2^	56.1±6.7 ^2^	***<0*.*001***^a^
Gender						0.27^c^
Female	11(55.0)	37(71.2)	32(51.6)	31(59.6)	21(65.6)	
Male	9(45.0)	15(28.8)	30(48.4)	21(40.4)	11(34.4)	
BMI (kg/m2)	23.2±2.8 ^2^^,^^3^^,^^4^^,^^5^	38.5±9.2 ^1^^,^^4^	36.2±7.3 ^1^	34.2±6.2 ^1^^,^^2^	35.8±8.0 ^1^	***<0*.*001***^a^
ALT (U/L)	---	41.0[27.0,64.5] ^4^	52.5[36.0,105.0]	71.5[48.0,105.0] ^2^^,^^5^	40.0[32.5,67.5] ^4^	***<0*.*001***^b^
Steatosis						***<0*.*001***^b^
None	---	0(0.0) ^3^^,^^4^	0(0.0) ^2^^,^^5^	2(3.8) ^2^	6(18.8) ^3^	
1–33%	---	33(63.5)	13(21.0)	12(23.1)	12(37.5)	
34–66%	---	11(21.2)	26(41.9)	26(50.0)	8(25.0)	
>66%	---	8(15.4)	23(37.1)	12(23.1)	6(18.8)	
Inflammation						***<0*.*001***^b^
No/minimal	---	41(78.8) ^3^^,^^4^^,^^5^	17(27.4) ^2^^,^^4^^,^^5^	4(7.7) ^2^^,^^3^	2(6.3) ^2^^,^^3^	
Mild	---	10(19.2)	35(56.5)	19(36.5)	9(28.1)	
Moderate	---	1(1.9)	10(16.1)	23(44.2)	16(50.0)	
Severe	---	0(0.0)	0(0.0)	6(11.5)	5(15.6)	
Ballooning						***<0*.*001***^b^
None	---	52(100.0) ^3^^,^^4^^,^^5^	26(41.9) ^2^^,^^4^^,^^5^	7(13.5) ^2^^,^^3^	5(15.6) ^2^^,^^3^	
Few	---	0(0.0)	30(48.4)	22(42.3)	10(31.3)	
Many	---	0(0.0)	6(9.7)	23(44.2)	17(53.1)	
Fibrosis						***<0*.*001***^b^
0	---	51(98.1) ^3^^,^^4^^,^^5^	19(30.6) ^2^^,^^4^^,^^5^	0(0.0) ^2^^,^^3^^,^^5^	0(0.0) ^2^^,^^3^^,^^4^	
1	---	1(1.9)	41(66.1)	0(0.0)	0(0.0)	
2	---	0(0.0)	2(3.2)	12(23.1)	0(0.0)	
3	---	0(0.0)	0(0.0)	40(76.9)	0(0.0)	
4	---	0(0.0)	0(0.0)	0(0.0)	32(100.0)	
NAS	---	1.8±0.90 ^3^^,^^4^^,^^5^	3.7±1.2 ^2^^,^^4^^,^^5^	4.8±1.5 ^2^^,^^3^	4.6±1.7 ^2^^,^^3^	***<0*.*001***^a^
WFA^+^ -M2BP (C.O.I)	0.44[0.38,0.63] ^4^^,^^5^	0.50[0.37,0.64] ^4^^,^^5^	0.66[0.49,0.94] ^4^^,^^5^	1.2[0.79,1.9] ^1^^,^^2^^,^^3^^,^^5^	2.4[1.5,4.2] ^1^^,^^2^^,^^3^^,^^4^	***<0*.*001***^b^

Statistics presented as Mean ± SD, Median [P25, P75] or N (column %).

p-values:

^*a*^ = ANOVA,

^*b*^ = Kruskal-Wallis test,

^*c*^ = Pearson’s chi-square test.

^1^: Significantly different from Healthy Control

^2^: Significantly different from NAFL

^3^: Significantly different from Early NASH

^4^: Significantly different from Fibrotic NASH

^5^: Significantly different from NASH Cirrhosis

Bonferroni correction was used for all pairwise post-hoc comparisons.

### WFA^+^ -M2BP levels correlate with NAFLD severity and its histological features

**Fig 1:**
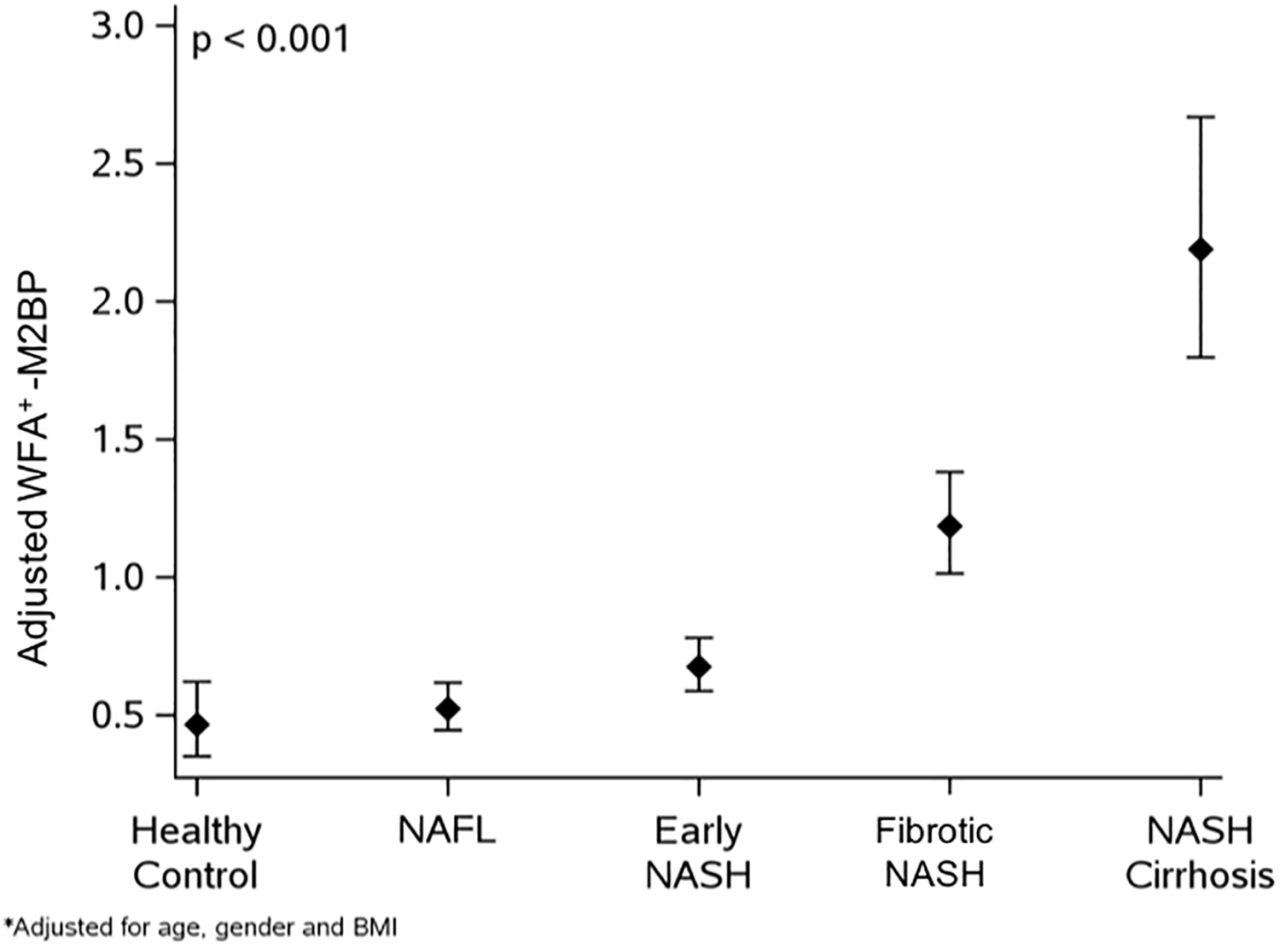
WFA^+^ -M2BP is increased in fibrotic NASH and NASH cirrhosis–Adjusted analysis. Patients with fibrotic NASH and NASH cirrhosis were found to have significantly higher WFA^+^ -M2BP levels than healthy controls, NAFL and early NASH (p<0.001). In addition, NASH cirrhosis had significantly higher WFA^+^ -M2BP levels than fibrotic NASH (p<0.001). There was no evidence of any difference between healthy controls, NAFL and early NASH (p>0.20).

There was a progressive stepwise increase in WFA^+^ -M2BP levels according to the presence of NAFLD and its histological severity, healthy controls = 0.44 [0.38,0.63], NAFL = 0.50 [0.37,0.64], early NASH = 0.66 [0.49,0.94], fibrotic NASH = 1.2 [0.79,1.9], and NASH cirrhosis = 2.4 [1.5,4.2]. WFA^+^ -M2BP level was found to be significantly increased in the fibrotic NASH and NASH cirrhosis groups compared to healthy controls and those with NAFL or early NASH after adjusting for age, gender and BMI (Fig 1), p value <0.001 as shown in Table 1. In addition, NASH cirrhosis patients had significantly higher WFA^+^ -M2BP levels than those with fibrotic NASH (p<0.001). There was no evidence of any difference between healthy controls, NAFL and early NASH (p>0.20).

Importantly, WFA^+^ -M2BP level had moderate correlation with inflammation [correlation coefficient rho (95% CI) = 0.45 (0.33, 0.55)], ballooning [0.45 (0.33, 0.55)] and NAFLD activity score [0.42 (0.30, 0.53)], p value < 0.001 for all. Moreover, WFA^+^ -M2BP correlated strongly with fibrosis stage with a correlation coefficient of 0.61 (0.52, 0.69), p value < 0.001 (Table 2). WFA^+^ -M2BP was found to be poorly correlated with ALT [rho (95% CI) = 0.17 (0.03, 0.30), p value = 0.016] and had no correlation with steatosis grade.

**Table 2:** Correlation between WFA^+^ -M2BP level and other severity measures.

Factor	rho (95% CI)	p-value
ALT	0.17 (0.03, 0.30)	***0*.*016***
Steatosis	0.03 (-0.11, 0.17)	0.71
Inflammation	0.45 (0.33, 0.55)	***<0*.*001***
Ballooning	0.45 (0.33, 0.55)	***<0*.*001***
Fibrosis	0.61 (0.52, 0.69)	***<0*.*001***
NAS	0.42 (0.30, 0.53)	***<0*.*001***

rho: Spearman’s correlation coefficient; CI: confidence interval

**Fig 2:**
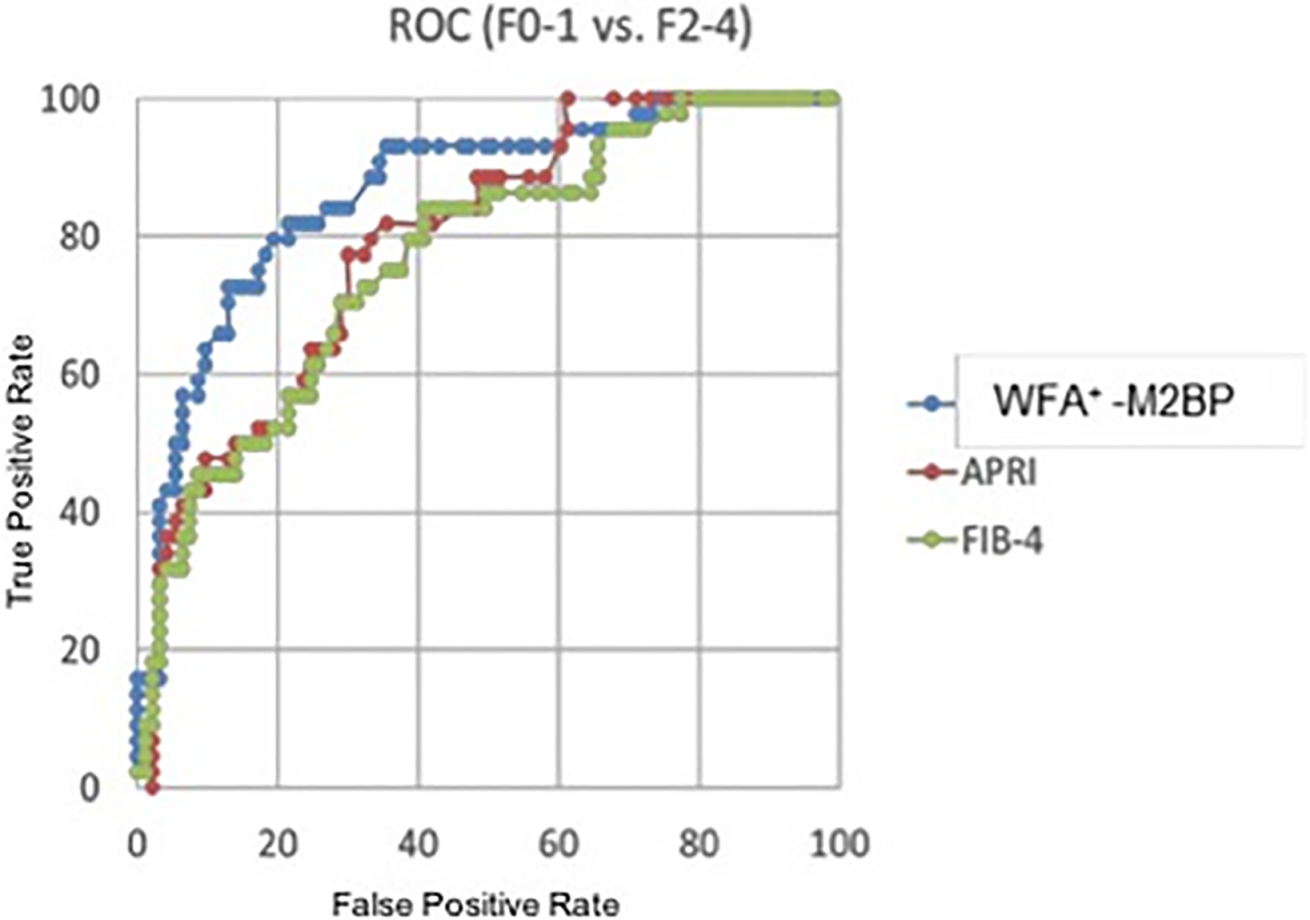
WFA^+^ -M2BP can discriminate fibrotic NASH & cirrhosis from NAFL & early NASH. The area under the ROC curve (AUC) of WFA^+^ -M2BP, APRI and FIB-4 were [0.866 (0.801, 0.931)], [0.792 (0.716, 0.868)] and [0.770 (0.687, 0.852)], respectively.

For ROC curve analysis, 137 of 198 samples from NAFLD patients which had a set of WFA^+^ -M2BP, age, ALT, AST and Platelets data were used. ROC curve analysis showed that WFA^+^ -M2BP can discriminate fibrotic NASH and NASH cirrhosis (F2-F4) from NAFL and early NASH (F0-F1) better than APRI and FIB-4 scores (Fig 2); The area under the ROC curve (AUC) of WFA^+^ -M2BP, APRI and FIB-4 were [0.866 (0.801, 0.931)], [0.792 (0.716, 0.868)] and [0.770 (0.687, 0.852)], respectively.

## Discussion

The main findings of this study are as follows: 1) In a large cohort of Western subjects, WFA^+^ -M2BP was a useful biomarker in predicting the presence of fibrotic NASH and NASH cirrhosis, 2) WFA^+^ -M2BP performed better than routinely used fibrosis scores to predict the presence of fibrotic NASH, 3) this novel biomarker correlated moderately with the presence of hepatocyte ballooning and inflammation and strongly with fibrosis stage confirming previous findings in Asian populations.

Fibrotic NASH is a serious condition that can progress to NASH cirrhosis and its complications and compared to earlier stages of the disease (NAFL and early NASH) is associated with increased hazard ratio for liver related mortality [15]. The presence of NASH cirrhosis has multiple implications including an increased risk for HCC and gastroesophageal varices. Therefore, patients with fibrotic NASH and NASH cirrhosis are considered as a high-priority for pharmacologic treatment and most clinical trials have focused on this patient population. Having a reliable serum-based biomarker that can identify patients with advanced disease has the potential to help clinicians in risk-stratifying their patients and researchers in decreasing the screen failure rate for pharmacologic trials.

Recent advances in the field of glycobiology have shown that changes in oligosaccharides occur in different human diseases making them attractive targets for biomarker development [16]. M2BP has been identified as one of the major glycoproteins secreted from injured hepatocytes. WFA^+^ -M2BP is a glycan structure isoform of M2BP and can be measured with a rapid, simple glycan-based immunoassay to quantify liver fibrosis in different chronic liver diseases [11, 17, 18]. The first study to assess the performance of WFA^+^ -M2BP in predicting disease severity in NAFLD was done by Abe et al. and included 289 Japanese patients with biopsy-proven disease. That study showed a clear association between WFA^+^ -M2BP level and the stage of fibrosis with an area under the ROC curve of 0.876 for predicting fibrosis ≥ stage2 [19]. More recently, several studies have shown the utility of this novel biomarker in predicting the presence of NASH and liver fibrosis within the NAFLD spectrum; however, all studies were done in Asian countries and to the best of our knowledge there are no studies in Western populations that may have different phenotypic and genotypic background [20, 21].

Our study has several strengths such as the inclusion of a large diverse cohort of patients with biopsy-proven disease and the availability of a healthy control group with no risk factors for NAFLD. We also divided the patients with NAFLD into four categories according to the recently published EASL guidelines which will help identify patients who need pharmacologic treatment the most, those with fibrotic NASH and NASH cirrhosis. Our study has a few limitations including the lack of liver biopsy or liver imaging in the healthy control group to ensure that there was no evidence of any fatty infiltration; however, these individuals did not have any of the classic risk factors for NAFLD. Second, this was a cross-sectional study with no longitudinal follow up to assess dynamic changes in WFA^+^ -M2BP levels with disease progression or regression. Finally, this study lacks an external validation group to confirm the accuracy of the proposed cutoff values in differentiating different disease stages.

In conclusion, in a large cohort of Western patients with the full spectrum of NAFLD, WFA^+^ -M2BP levels predicted the presence of advanced disease and correlated strongly with fibrosis stage. WFA^+^ -M2BP appears to be a promising biomarker for NAFLD severity that will potentially help risk-stratify patients for enrollment in current clinical trials and could possibly replace liver biopsy in the future. With further validation, WFA^+^ -M2BP could also be integrated into a clinical algorithm to help primary care providers in determining the need for specialty referrals for advanced NAFLD management. Further studies are needed to assess dynamic changes in this biomarker in response to NAFLD treatments and its prognostic utility in predicting liver-related outcomes.

## Supporting information

S1 Dataset Minimal dataset. (XLSX)

## Abbreviations

NAFLD: nonalcoholic fatty liver disease
NASH: nonalcoholic steatohepatitis
WFA^+^ -M2BP: *Wisteria floribunda* agglutinin-positive Mac-2-binding protein
