# Correction: Serum *Wisteria floribunda* agglutinin-positive Mac-2-binding protein levels predict the presence of fibrotic nonalcoholic steatohepatitis (NASH) and NASH cirrhosis

**DOI:** 10.1371/journal.pone.0205541

**Published:** 2018-10-04

**Authors:** Naim Alkhouri, Casey Johnson, Leon Adams, Sachiko Kitajima, Chikayuki Tsuruno, Tracey L. Colpitts, Kazuki Hatcho, Eric Lawitz, Rocio Lopez, Ariel E. Feldstein

The seventh author's name is spelled incorrectly. The correct name is: Ariel E. Feldstein
